# Supplementary material for: Carbon nitride supported Fe2 cluster catalysts with superior performance for alkene epoxidation
Source: Nat Commun. 2018 Jun 15;9:2353. doi: 10.1038/s41467-018-04845-x (PMC6003949; doi:10.1038/s41467-018-04845-x)
Supplement: Supplementary file 1 — Supplementary Information [file 41467_2018_4845_MOESM1_ESM.docx]

**Carbon Nitride** **Supported** **Fe_2_ Cluster Catalysts with Superior Performance for** **Alkene Epoxidation**

Supplementary Information

tian et al.

**Supplementary Methods**

**Chemicals**

All chemicals were used as received without any further purification. Bis(dicarbonylcyclopentadienyliron) (99.9%), Allylpalladium(II) chloride dimer (98%), Bis(1,5-cyclooctadiene)diiridium(I) dichloride (99%), Iron(III) acetylacetonate, Ruthenium acetylacetonate, Palladium acetylacetonate, Rhodium acetylacetonate, Platinum acetylacetonate and HAuCl_4_▪4H_2_O were purchased from Alfa Aesar. Cyanamide (98%), *trans*-Stilbene oxide (99%) and Silica gel, for chromatography (0.035-0.070 mm, 60 Å) were purchased from Sigma-Aldrich. N,N-Dimethylformamide (99%), N,N-Dimethylacetamide (99.9%), methanol (99%) and n-hexane (99.9%) were purchased from Sinopharm Chemical Reagent Co. Ltd. (Shanghai, China).

**Synthesis of single-atom Fe_1_/mpg-C_3_N_4_**

In a typical synthesis of Fe_1_/mpg-C_3_N_4_, 20 mg iron porphyrin and 500 mg mpg-C_3_N_4_ were dissolved in 100 mL DMF under stirring at room temperature for 24h. The product was separated by centrifugation and then washed with DMF and methanol thoroughly. The as-prepared powder was pyrolyzed at 400 ^o^C for 2h under flowing mixture of 5% H_2_/Ar atmosphere, and during this period, the ligands of iron porphyrin were found to be removed. The Fe loading is 0.18% determined by the ICP-AES analysis.

**Synthesis of Fe/GO**

In a typical synthesis of Fe/GO, 2mg Bis(dicarbonylcyclopentadienyliron) (Fe_2_O_4_C_14_H_10_) and GO (200mg) were dissolved in the DMF (100 mL) under stirring at room temperature for 24h. The product was separated by centrifugation and washed subsequently with DMF then methanol thoroughly. The as-prepared powder was transferred into a ceramic both and then placed into a tube furnace maintaining 300 ^o^C for 2h under flowing mixture of 5% H_2_/Ar atmosphere with a heating rate of 5 ^o^C min-1. The Fe loading is 0.17% determined by ICP-AES analysis.

**Synthesis of metal nanoparticles**

In a typical preparation of Fe nanocrystals, 20mg Iron(III) acetylacetonate was dissolved in OAm (10 mL) at 250 ^o^C under vigorous stirring for 10 min. Then, a solution of borane-tert-butylamine (100 mg, 1.15 mmol) in OAm (2 mL) was added quickly into the previous solution, and the reaction mixture immediately turned black. After 2 min, the flask was heated to 270 ^o^C for a further 1 h. After cooling to room temperature, the solution was washed with ethanol and then dispersed in cyclohexane for future use. In the same procedure, other mental nanoparticles were successfully synthesized, except the Iron(III) acetylacetonate was replaced by the corresponding metal acetylacetonate and the reaction temperature was changed. The temperature of synthesis of Ru NPs, Rh NPs, Pd NPs and Pt NPs is 250 ^o^C, 200 ^o^C, 160 ^o^C and 120 ^o^C, respectively.

**Synthesis of metal nanoparticles/mpg-C_3_N_4_**

In a typical synthesis of Fe nanoparticles/mpg-C_3_N_4_, Fe nanoparticles (2 mg) and mpg-C_3_N_4_ (500mg) were dissolved in the mixture of ethanol (100 mL) and n-hexane (100 mL) under stirring at room temperature for 24h. The product was separated by centrifugation at 10000 rpm for 5min and washed subsequently with n-hexane for once time, then washed with methanol for once and finally dried under vacuum at room temperature. The as-prepared powder was transferred into a ceramic both and then placed into a tube furnace maintaining 300 ^o^C for 2h under flowing mixture of 5% H_2_/Ar atmosphere with a heating rate of 5 ^o^C min^-1^. The Fe loading is 0.18% determined by ICP-AES analysis. In the same procedure, other mental nanoparticles were successfully synthesized, except the Fe nanoparticles were replaced by the corresponding metal nanoparticle. The loading of Ru, Rh, Pd and Pt is 0.21%, 0.23%, 0.19% and 0.25% determined by ICP-AES analysis.

Synthesis of Au nanoparticles/mpg-C_3_N_4_

In a typical synthesis of Au nanoparticles/mpg-C_3_N_4_, 10 mg HAuCl_4_▪4H_2_O and mpg-C_3_N_4_ (200mg) were dissolved in the H_2_O (50 mL) under stirring at room temperature for 12h. The product was separated by centrifugation and washed subsequently with DMF then methanol thoroughly. The as-prepared powder was pyrolyzed at 200 ^o^C for 2h under flowing mixture of 5% H_2_/Ar atmosphere. The Au loading is 1.9% determined by ICP-AES analysis.

**Synthesis of Pd_2_/mpg-C_3_N_4_**

In a typical synthesis of Pd_2_/mpg-C_3_N_4_, (η_3_-C_3_H_5_)_2_Pd_2_Cl_2_ (5mg) and mpg-C_3_N_4_ (500mg) were dissolved in the DMF (100 mL) under stirring at room temperature for 24h. The product was separated by centrifugation at 10000 rpm for 5min and washed subsequently with DMF for once time, then washed with methanol for once and finally dried under vacuum at room temperature. The as-prepared powder was transferred into a ceramic both and then placed into a tube furnace maintaining 300 ^o^C for 2h under flowing Ar atmosphere with a heating rate of 5 ^o^C min^-1^. The Pd loading is 0.19% determined by ICP-AES analysis.

**Synthesis of Ir_2_/mpg-C_3_N_4_**

In a typical synthesis of Ir_2_/mpg-C_3_N_4_, C_16_H_24_Cl_2_Ir_2_ (5mg) and mpg-C_3_N_4_ (500mg) were dissolved in the DMF (100 mL) under stirring at room temperature for 24h. The product was separated by centrifugation at 10000 rpm for 5min and washed subsequently with DMF for once time, then washed with methanol for once and finally dried under vacuum at room temperature. The as-prepared powder was transferred into a ceramic both and then placed into a tube furnace maintaining 300 ^o^C for 2h under flowing Ar atmosphere with a heating rate of 5 ^o^C min^-1^. The Ir loading is 0.22% determined by ICP-AES analysis.

**Characterization**

Powder X-ray diffraction patterns of samples were recorded on a Rigaku D/max 2500Pc X-ray powder diffractometer using a Cu Kα radiation (λ=0.15418nm). TEM images were recorded on a Hitachi-7700 worked at 100 kV. HRTEM images were obtained by a FEI Tecnai G2 F20 S-Twin HRTEM working at 200 kV. Atomic resolution HAADF-STEM images were imaged by using a Titan 80-300 scanning/transmission electron microscope operater at 300 kV, equipped with a probe spherical aberration corrector. Inductively coupled optical emission spectroscopy (ICP-OES) was performed on Thermo Fisher IRIS Intrepid Ⅱ. Gas chromatography (GC) was performed on Thermo Fisher scientific Trace 1300 with a flame ionization detector and Gas chromatography mass spectrometry (GC-MS) was performed on Thermo Fisher scientific ISQ system.

**Supplementary Figures and Tables**


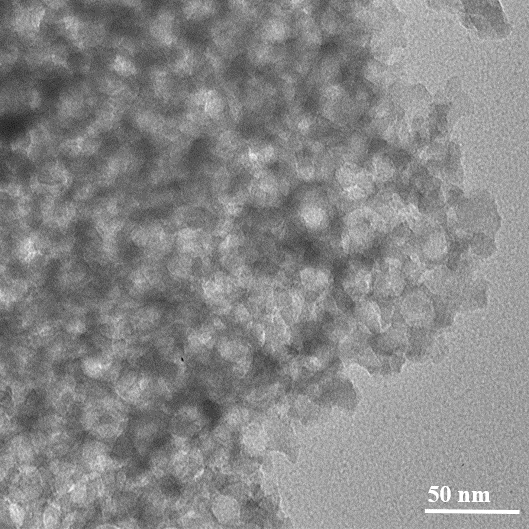


**Supplementary Figure 1**. TEM of mpg-C_3_N_4_.





**Supplementary Figure 2**. XRD patterns of the as-prepared mpg-C_3_N_4_ (black) and Fe_2_/mpg-C_3_N_4_ (red).





**Supplementary Figure 3**. IR patterns of the as-prepared mpg-C_3_N_4_ (black) and Fe_2_/mpg-C_3_N_4_ (red).


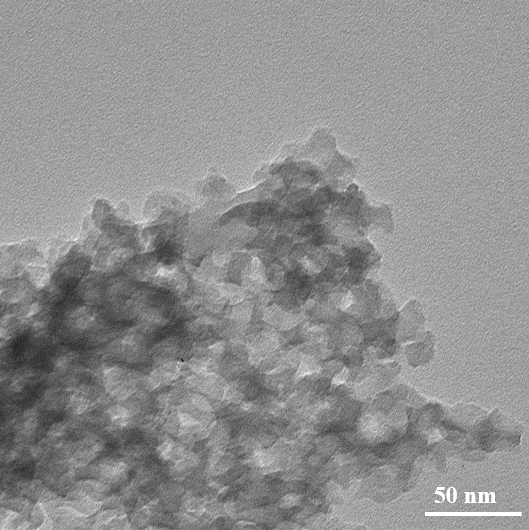


**Supplementary Figure 4**. TEM of Fe_2_/mpg-C_3_N_4_.


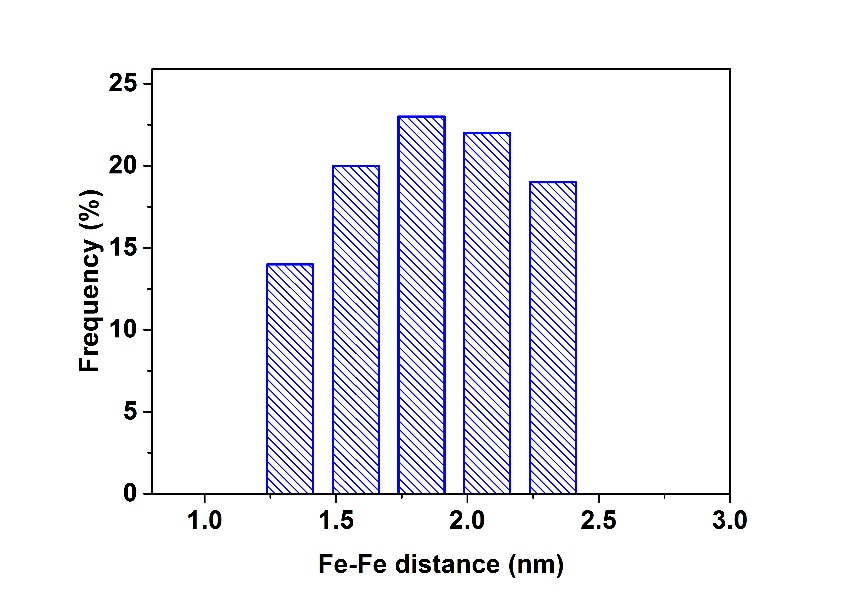


**Supplementary Figure 5.** Statistical Fe–Fe distance in the observed Fe_2_ clusters.


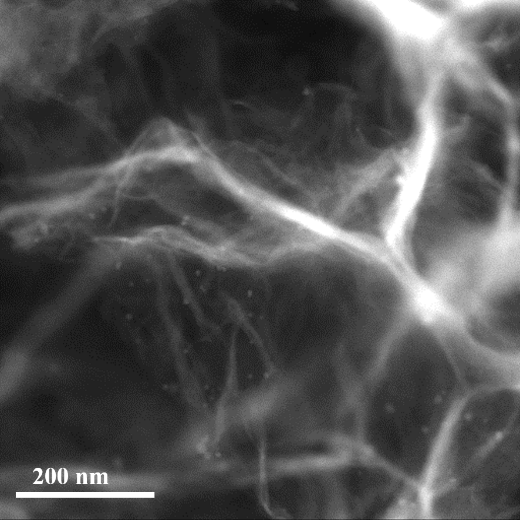


**Supplementary Figure 6.** HAADF-STEM images of the product using the GO as support.


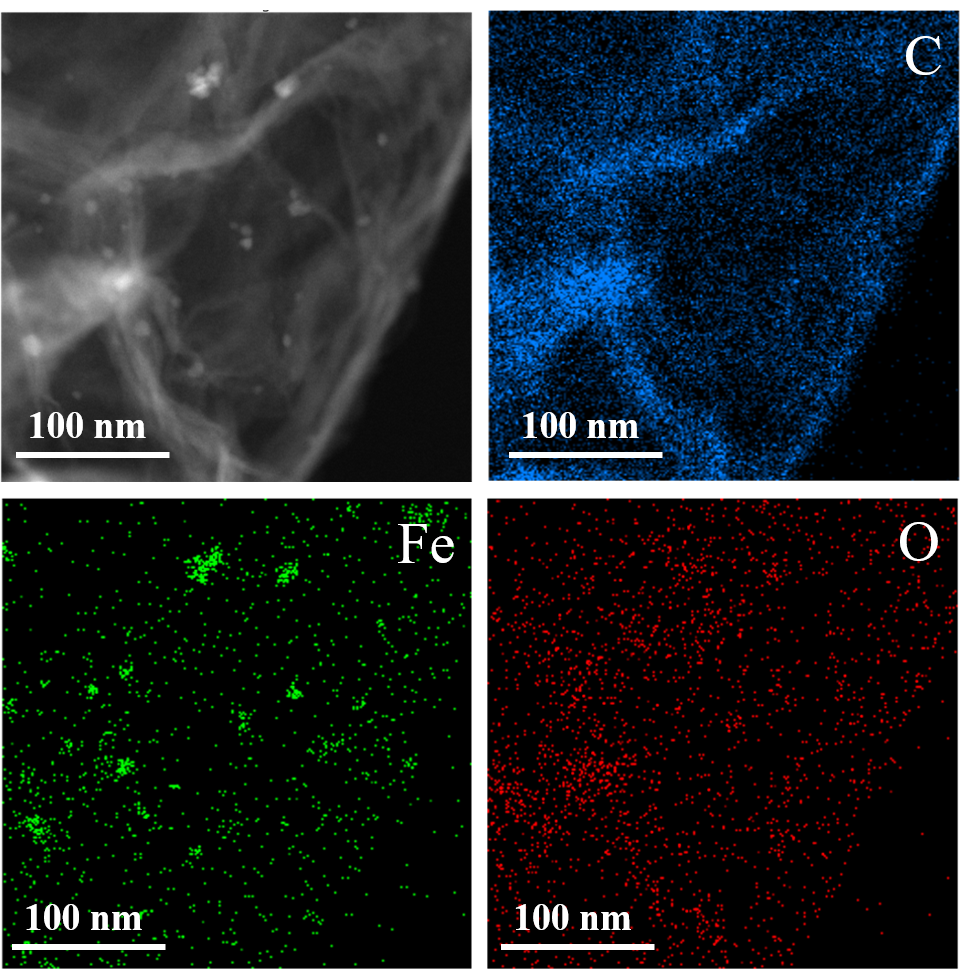


**Supplementary Figure 7.** Corresponding element maps showing distributions of Fe (green), N(red), C (blue), respectively.


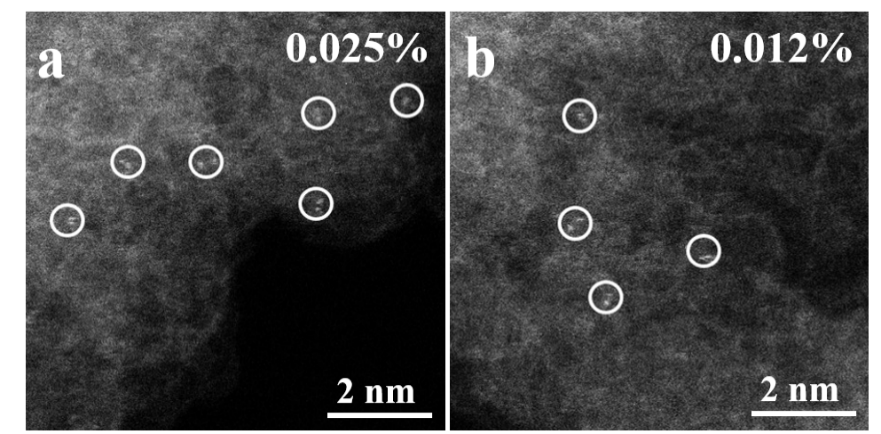


**Supplementary Figure 8.** AC HAADF-STEM images of Fe_2_/mpg-C_3_N_4_. (left: the loading Fe is 0.025wt%; right: the loading Fe is 0.012wt%).





**Supplementary Figure 9.** The TOF-SIMS spectrum around *m/z* 111.9 signals of Fe_2_/mpg-C_3_N_4_, Fe_2_ precursor and mpg-C_3_N_4_. The obvious fragment signal in Fe_2_/mpg-C_3_N_4_ was detected at *m/z* 111.88 in positive ion mode, which was consistent with observation of Fe_2_^+^ from Fe_2_ precursor. In addition, the *m/z* 111.88 signal (Fe_2_^+^) of mpg-C_3_N_4_ sample was not detected. The results were a powerful proof to further confirm the presence of Fe_2_ cluster species in Fe_2_/mpg-C_3_N_4_ sample.


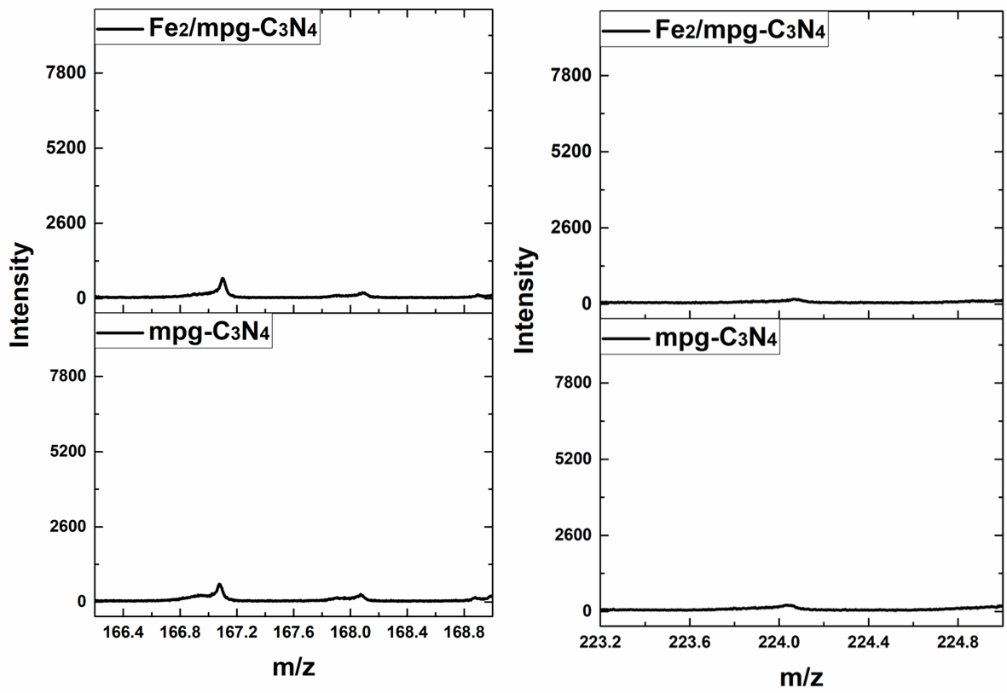


**Supplementary Figure 10.** The TOF-SIMS spectrum around Fe_3_^+^ and Fe_4_^+^ signals of Fe_2_/mpg-C_3_N_4_, and mpg-C_3_N_4_. No fragment signals in Fe_2_/mpg-C_3_N_4_ was detected at around *m/z* of Fe_3_^+^ and Fe_4_^+^. The results were a powerful proof to further confirm no larger Fe clusters species, such as Fe_3_ or Fe_4_, in Fe_2_/mpg-C_3_N_4_ sample.


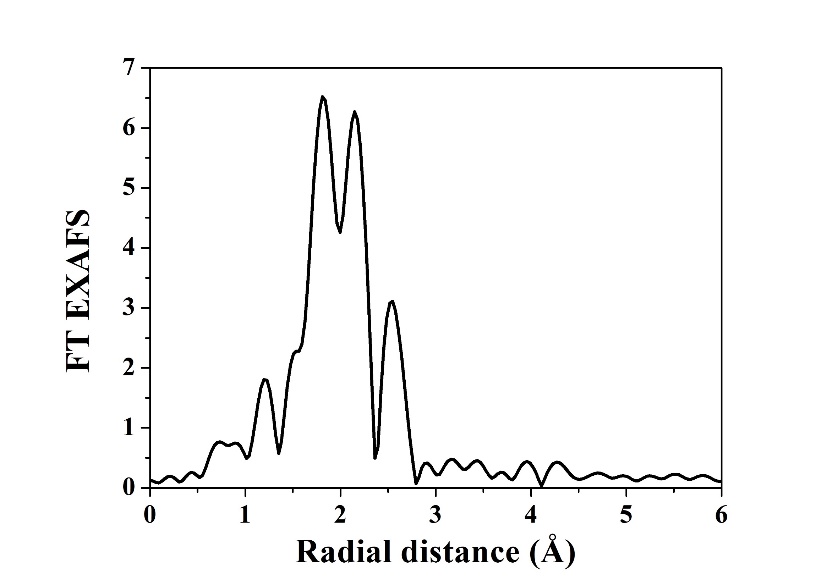


**Supplementary Figure 11.** The Fourier transform (FT) of EXAFS spectra at the Fe k-edge of Fe_2_ precursor.

**Supplementary Table 1**. Structural parameters of Fe_2_-C_3_N_4_ extracted from the EXAFS fitting. (S_0_^2^=0.85)

| Sample | Scattering pair | CN | R(Å) | σ^2^(10^-3^Å^2^) | ΔE_0_(eV) | R factor |
| --- | --- | --- | --- | --- | --- | --- |
| Fe_2_-C_3_N_4_ | Fe- N(O)_1_ | 0.8 | 1.71 | 4.7 | -1.0 | 0.0071 |
|  | Fe-N(O)_2_ | 3.0 | 2.00 | 8.2 |  |  |
|  | Fe-Fe | 1.2 | 2.43 | 4.1 | -2.5 |  |
| Fe foil | Fe-Fe_1_ | 8* | 2.47 | 5.8 | 1.5 | 0.0025 |
|  | Fe-Fe_2_ | 6* | 2.84 | 7.2 |  |  |

S_0_^2^ is the amplitude reduction factor; CN is the coordination number; R is interatomic distance (the bond length between central atoms and surrounding coordination atoms); σ^2^ is Debye-Waller factor (a measure of thermal and static disorder in absorber-scatterer distances); ΔE_0_ is edge-energy shift (the difference between the zero kinetic energy value of the sample and that of the theoretical model). R factor is used to value the goodness of the fitting.

* This value was fixed during EXAFS fitting, based on the known structure of Fe foil.

Error bounds that characterize the structural parameters obtained by EXAFS spectroscopy were estimated as N ± 20%; R ± 1%; σ^2^ ± 20%; ΔE_0_ ± 20%.


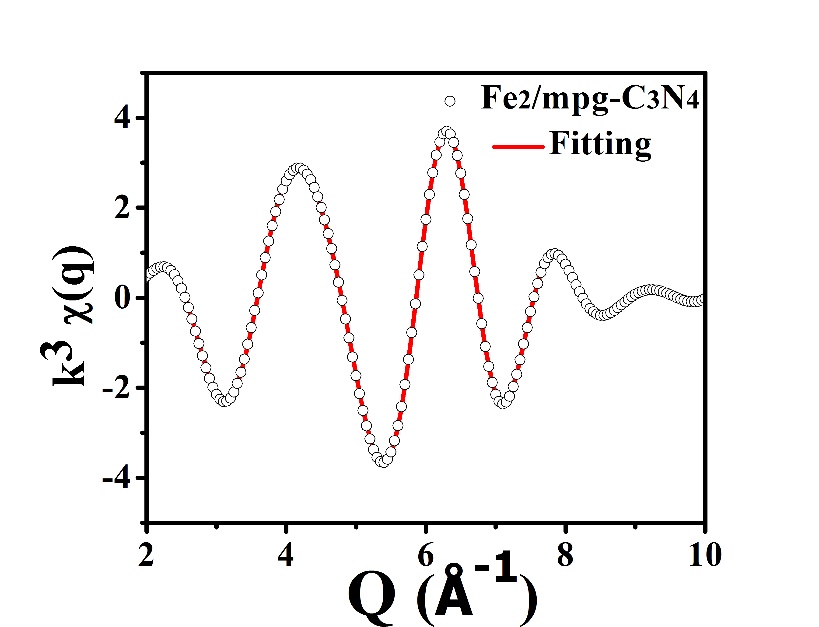


**Supplementary Figure 12.** EXAFS spectrum fitting curves of Fe_2_/mpg-C_3_N_4_ Powder at q space.


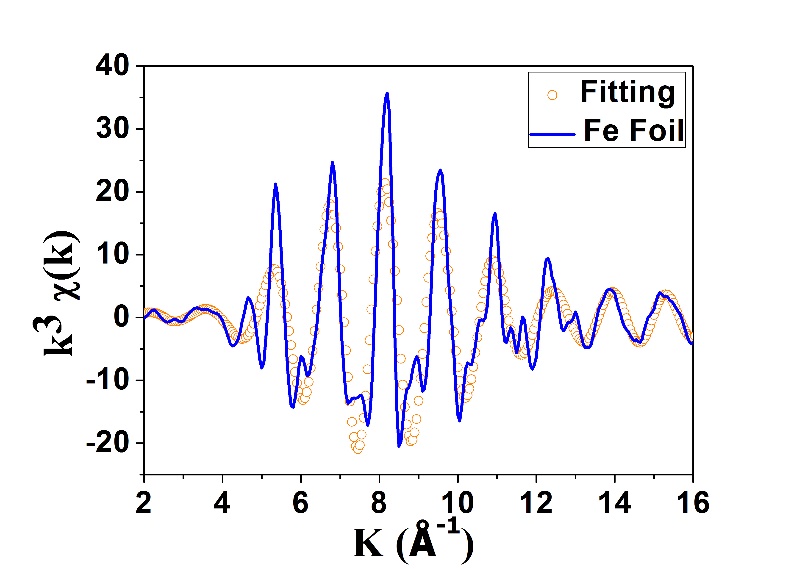


**Supplementary Figure 13.** EXAFS spectrum fitting curves of Fe foil at k space.


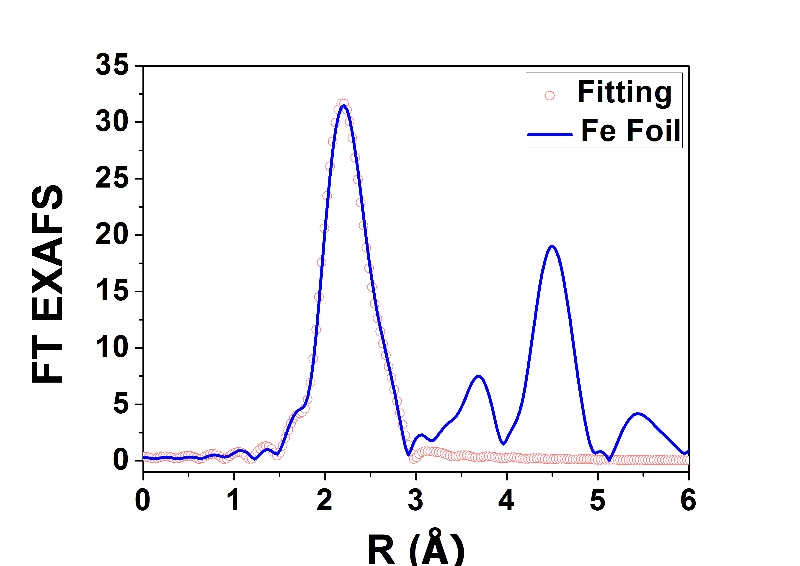


**Supplementary Figure 14.** EXAFS spectrum fitting curves of Fe foil at R space.


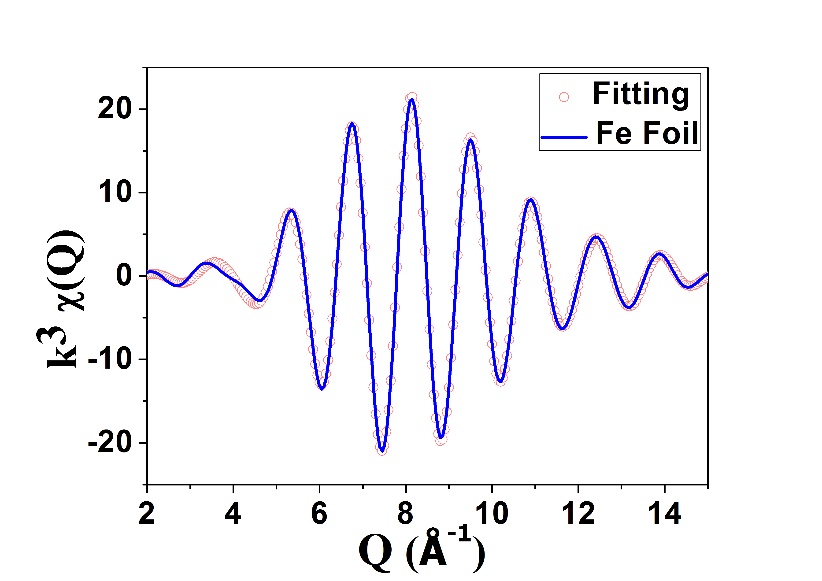


**Supplementary Figure 15.** EXAFS spectrum fitting curves of Fe foil at q space.


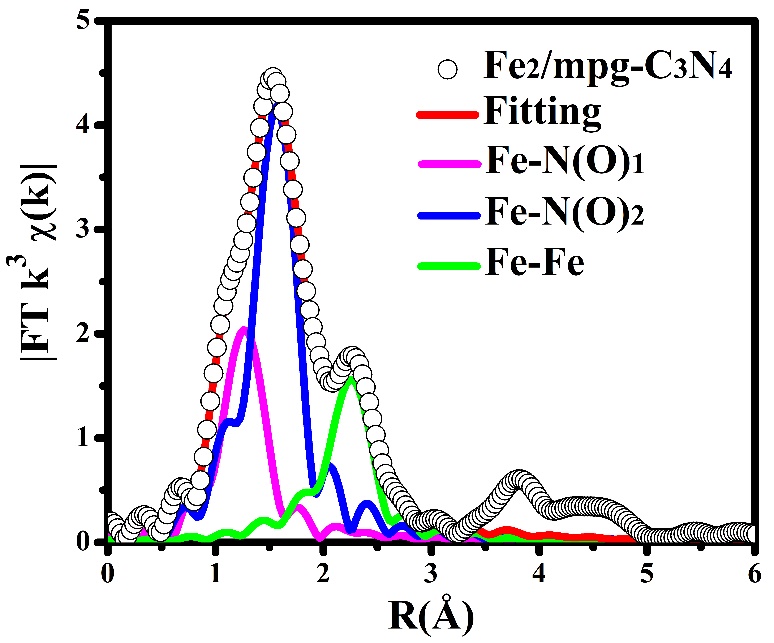


**Supplementary Figure 16.** EXAFS fitting curves for Fe_2_/mpg-C_3_N_4_.


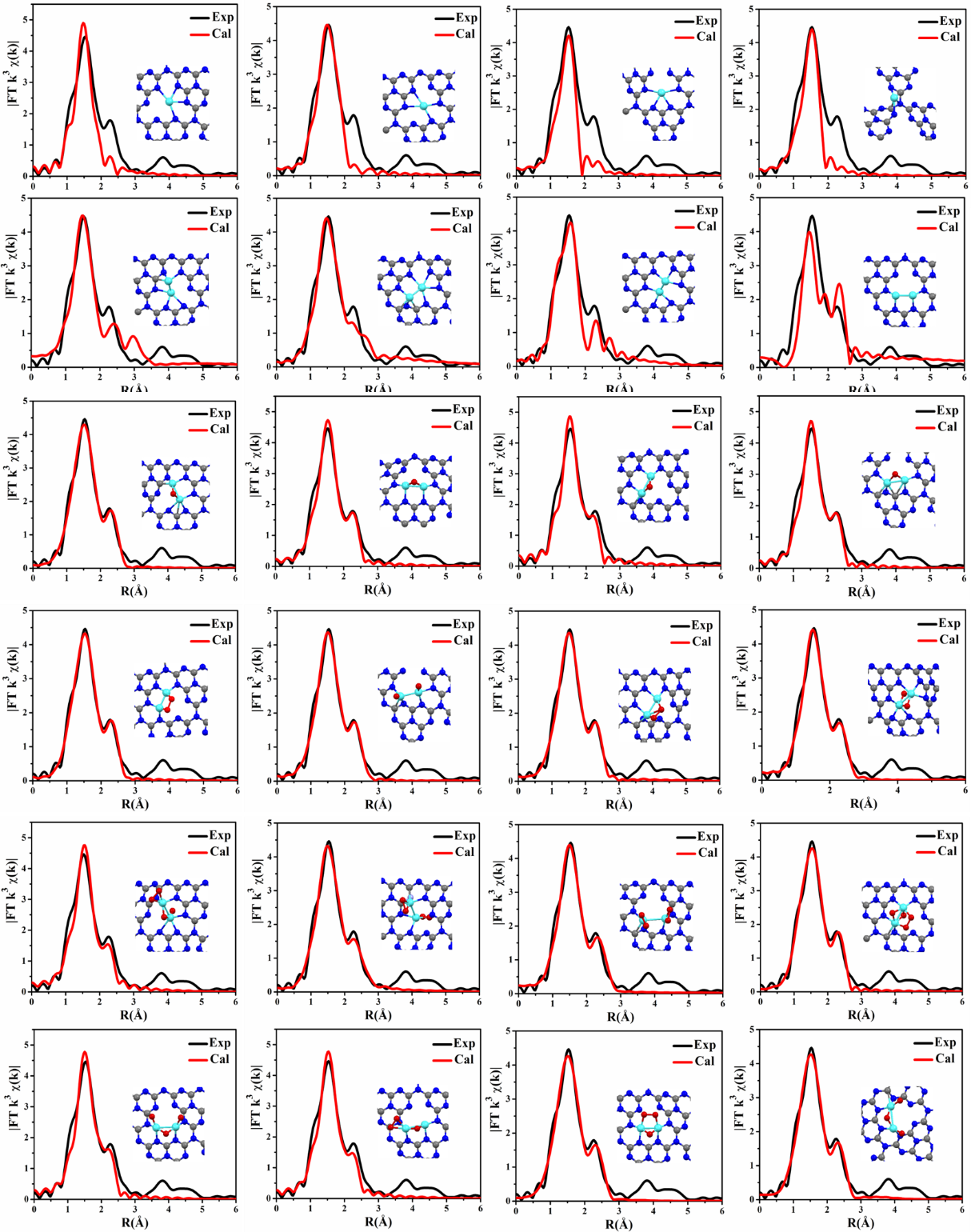


**Supplementary Figure 17.** Comparison between the Fe K-edge EXAFS experimental spectrum (solid black line) and the theoretical spectrum (solid red line) calculated with others different structures.


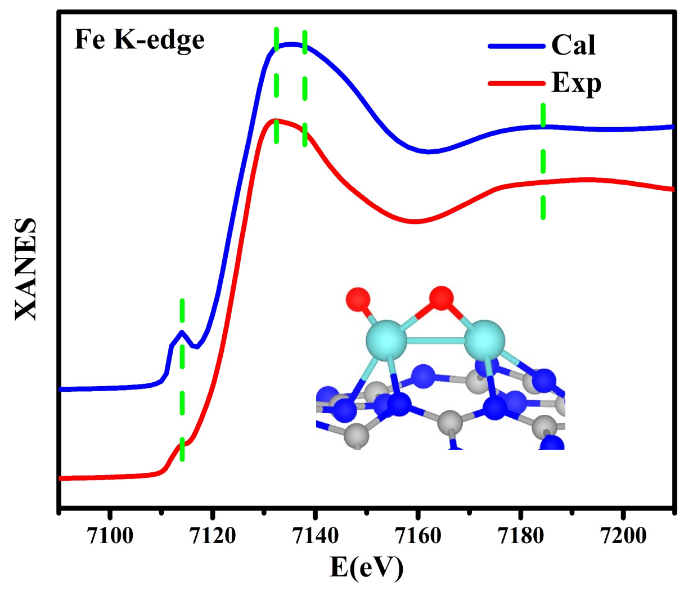


**Supplementary Figure 18.** Comparison between the Fe K-edge XANES experimental spectrum (solid red line) and the theoretical spectrum (solid blue line) calculated with the inset structure.


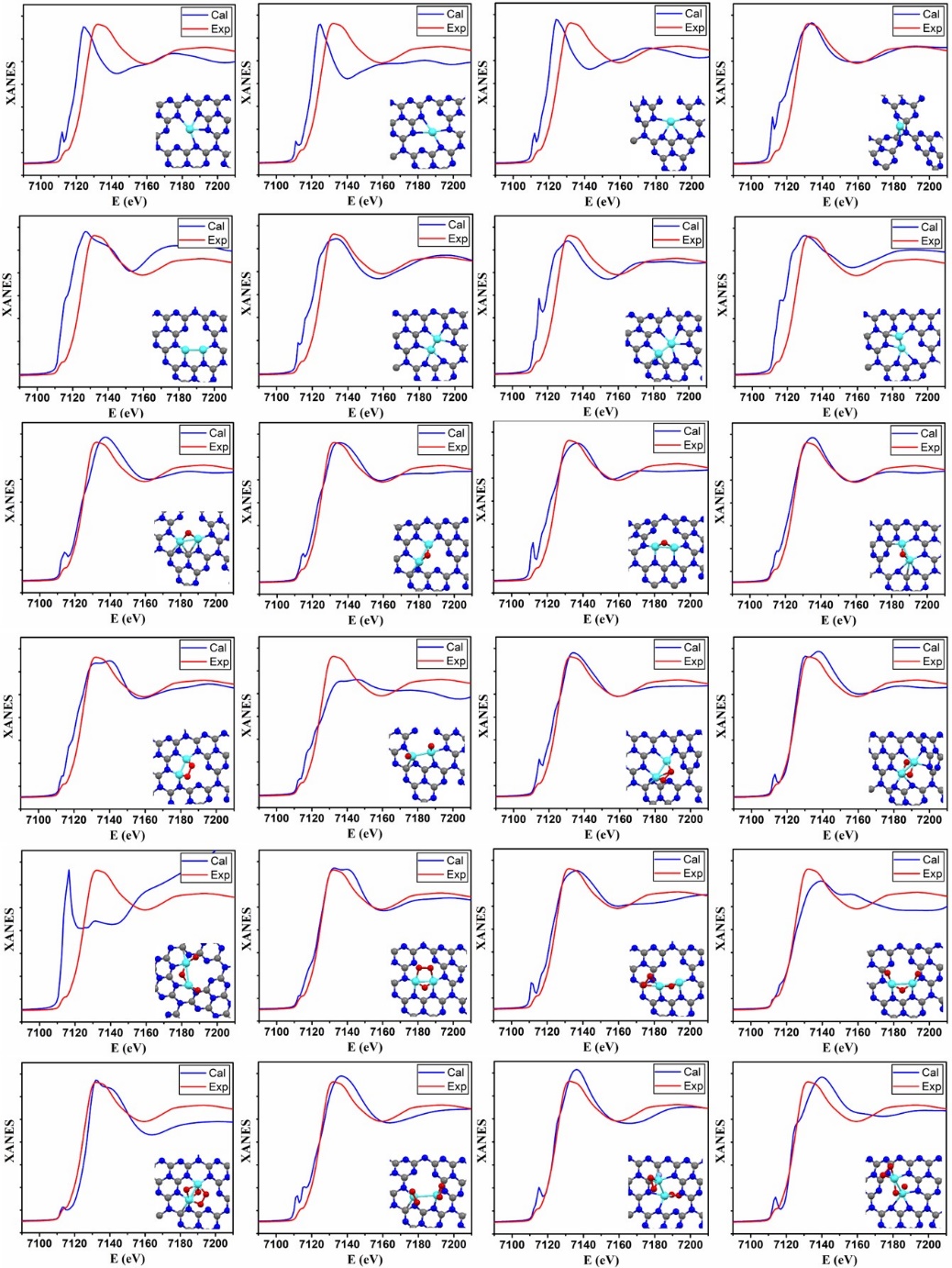


**Supplementary Figure 19.** Comparison between the Fe K-edge XANES experimental spectrum (solid red line) and the theoretical spectrum (solid blue line) calculated with others different structures.

The Fe K-edge theoretical XANES calculations were carried out with the FDMNES code in the framework of real-space full multiple-scattering (FMS) scheme using Muffin-tin approximation for the potential.^1-3^ The energydependent exchange-correlation potential was calculated in the real Hedin-Lundqvist scheme, and then the spectra convoluted using a Lorentzian function with an energy-dependent width to account for the broadening due both to the core–hole width and to the final state width.


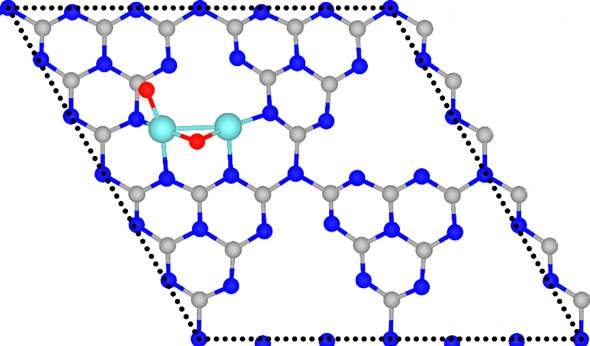


**Supplementary Figure 20**. Top view of the Fe_2_@mpg-C_3_N_4_ catalyst displayed as a ball-and-stick model. The carbon, nitrogen, oxygen, and iron atoms are depicted in gray, blue, red, and cyan, respectively. The dotted rhombus represents the 2×2 g-C_3_N_4_ unit cell.


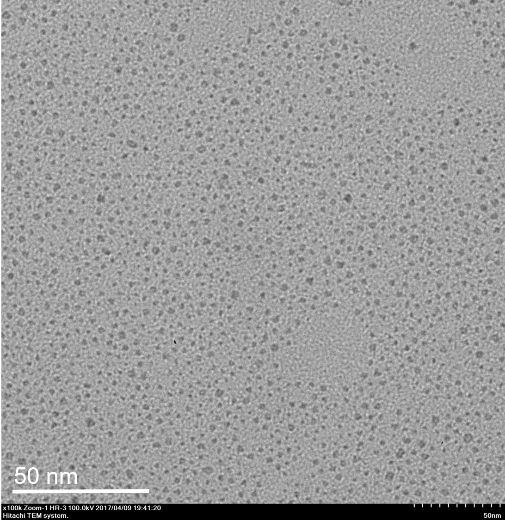


**Supplementary Figure 21.** The TEM image of Fe NPs.


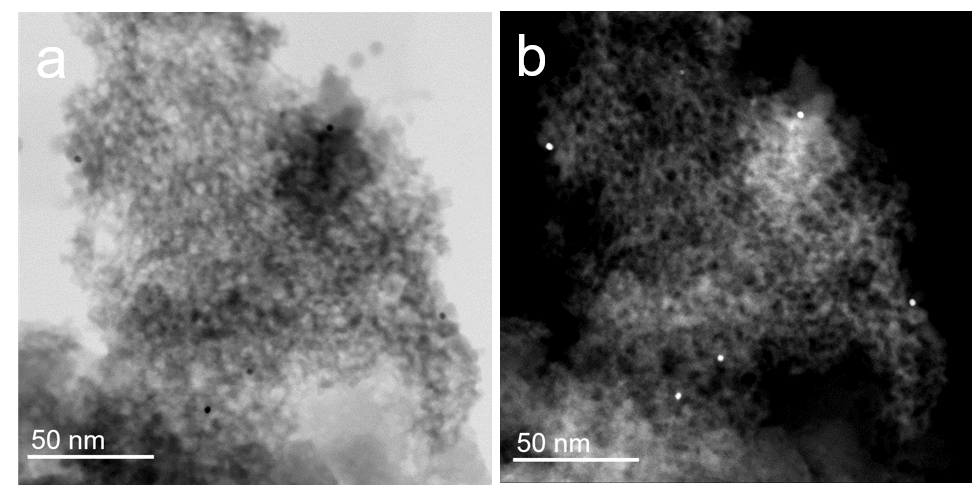


**Supplementary Figure 22.** The STEM image of Fe nanoparticles/C_3_N_4_.


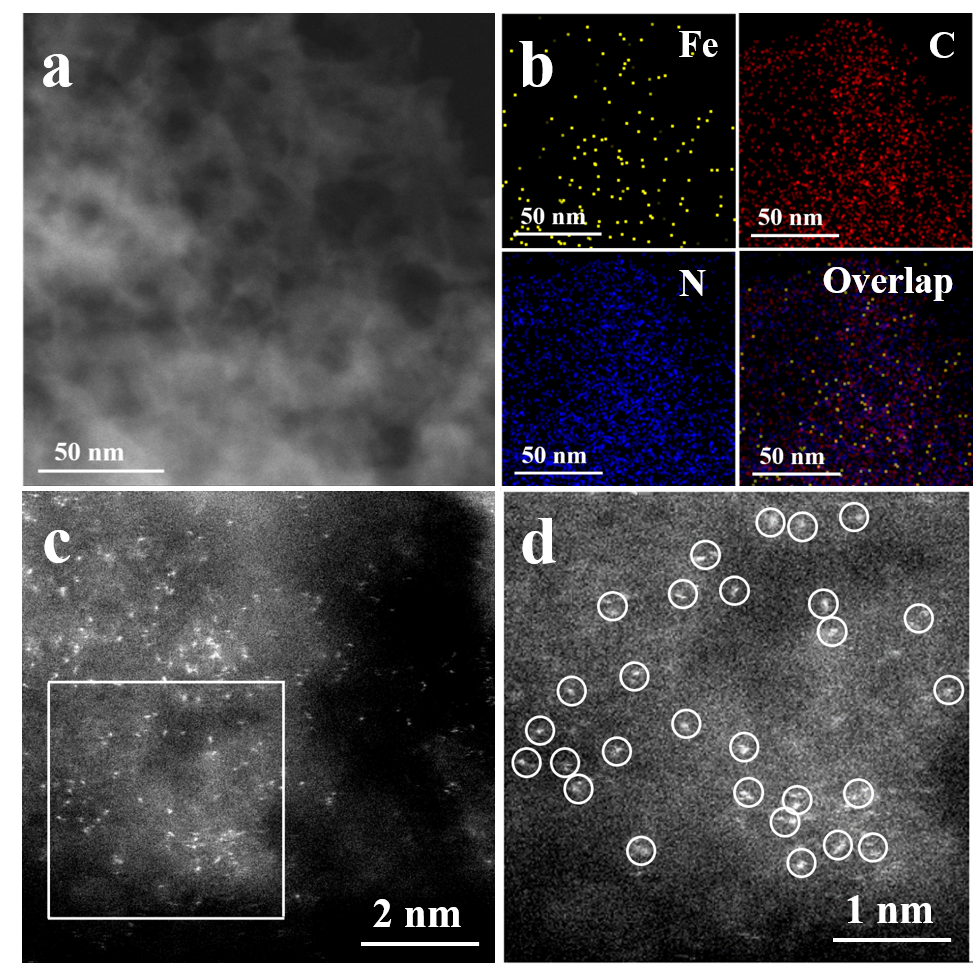


**Supplementary Figure 23.** (a) HAADF-STEM images of Fe_1_/mpg-C_3_N_4_. (b) corresponding element maps showing distributions of Fe (green), N(red), C (blue), respectively. (c) AC HAADF-STEM images of Fe_1_/mpg-C_3_N_4_. (d) Magnified AC HAADF-STEM images of Fe_1_/mpg-C_3_N_4_.


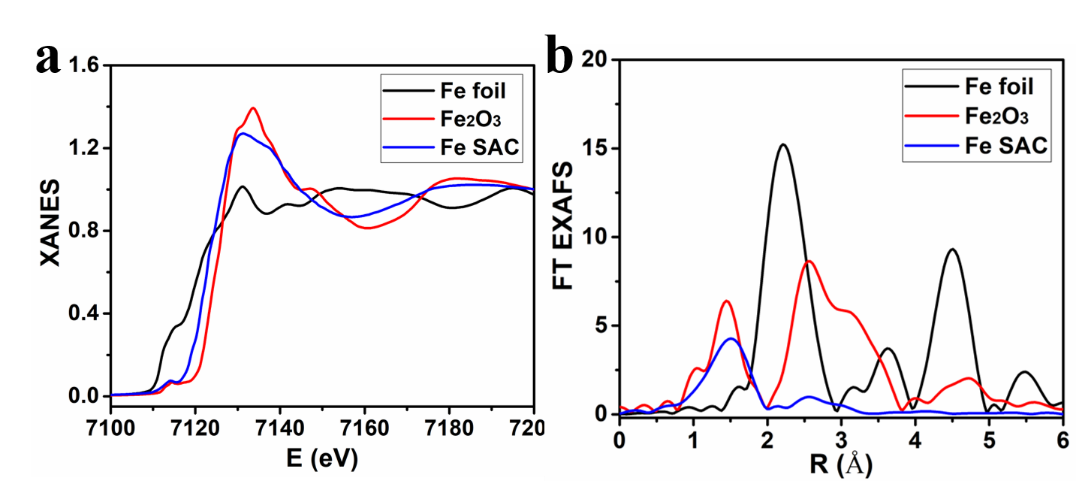


**Supplementary Figure 24.** (a) XANES spectra at the Fe k-edge of Fe_1_/mpg-C_3_N_4_, Fe_2_O_3_, and Fe foil. (b) Fourier transform (FT) at the Fe k-edge of Fe_1_/mpg-C_3_N_4_, Fe_2_O_3_ and Fe foil.

**Supplementary** **Table 2**. Catalytic epoxidation of trans -stilbene by Fe_2_/mpg-C_3_N_4_ and different noble metal nanoparticles catalysts.

| Catalyst | Conversion (%) | Selectivity (%) |
| --- | --- | --- |
| Fe_2_/mpg-C_3_N_4_ | 91 | 93 |
| Au NPs/mpg-C_3_N_4_  Ru NPs/mpg-C_3_N_4_  Rh NPs/mpg-C_3_N_4_  Pd NPs/mpg-C_3_N_4_  Pt NPs/mpg-C_3_N_4_ | 35  28  trace  trace  17 | 83  87  -  -  90 |


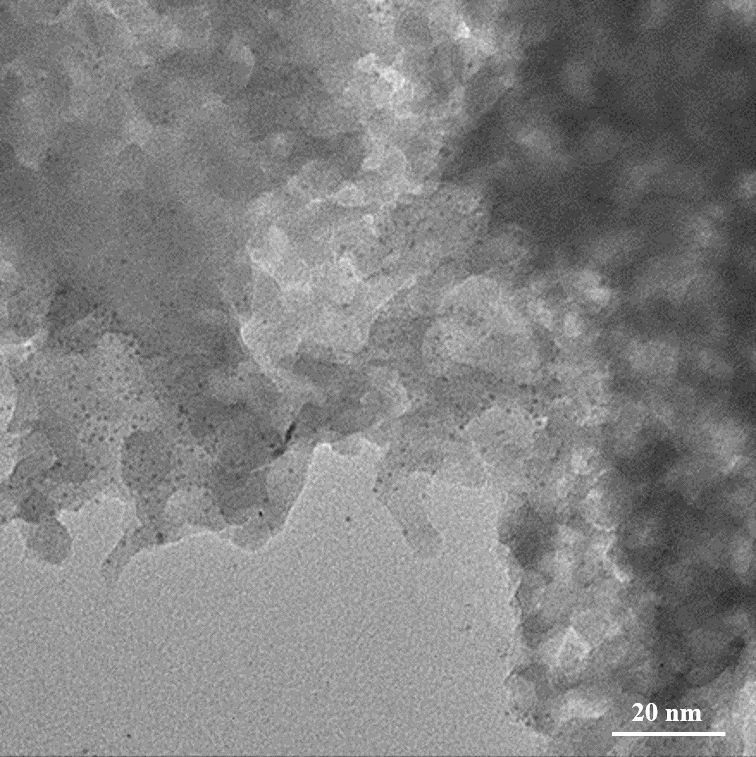


**Supplementary Figure 25.** TEM image of Ru nanoparticles.


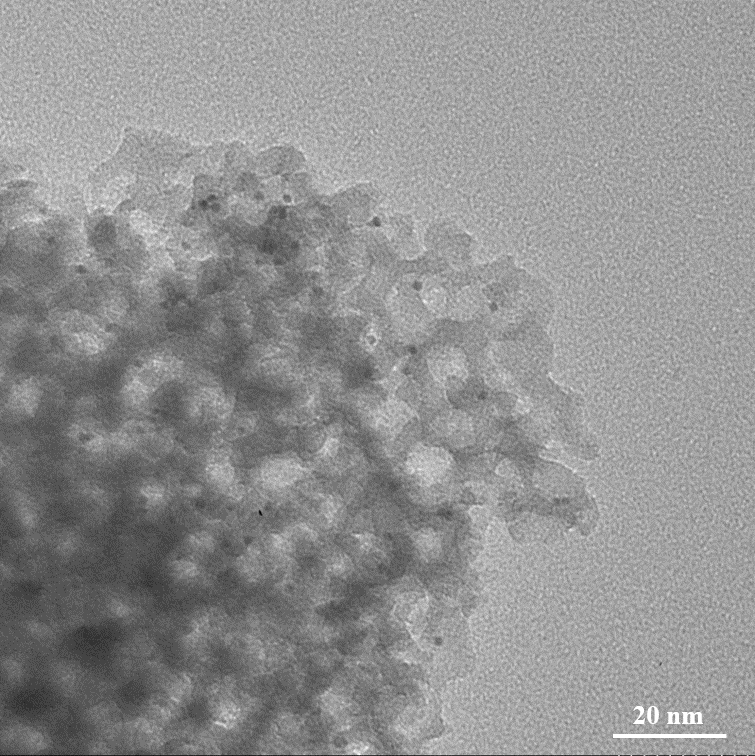


**Supplementary Figure 26.** TEM image of Rh nanoparticles.


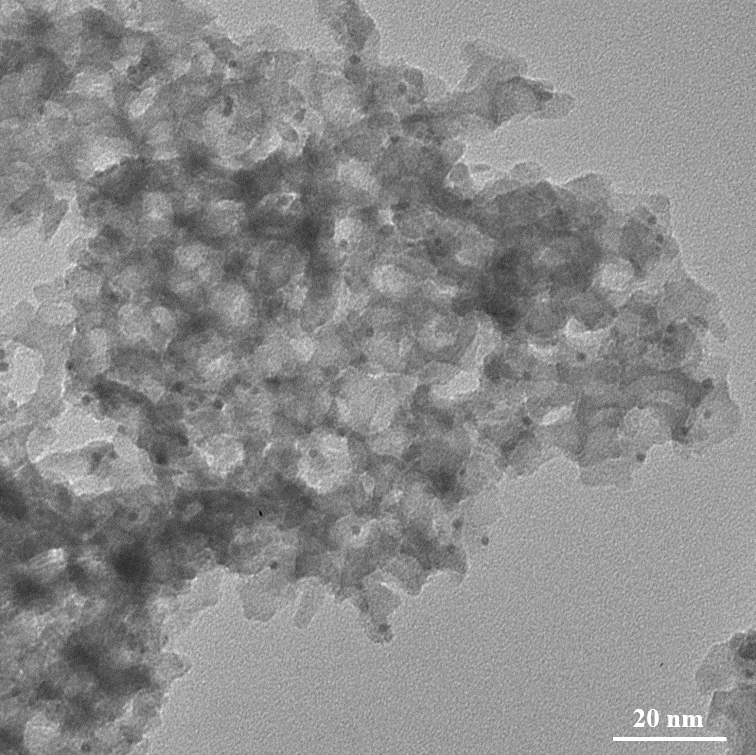


**Supplementary Figure 27.** TEM image of Pd nanoparticles.


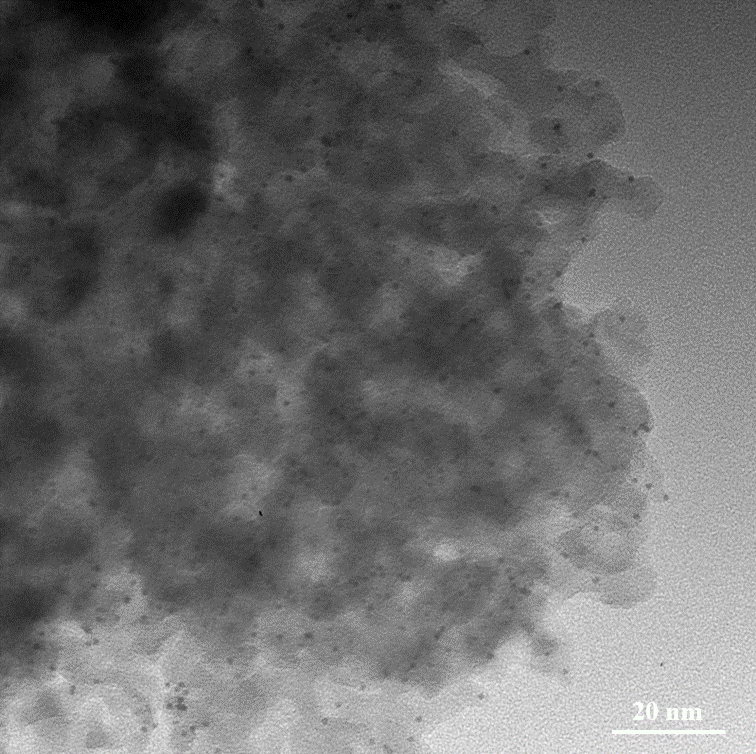


**Supplementary Figure 28.** TEM image of Pt nanoparticles.


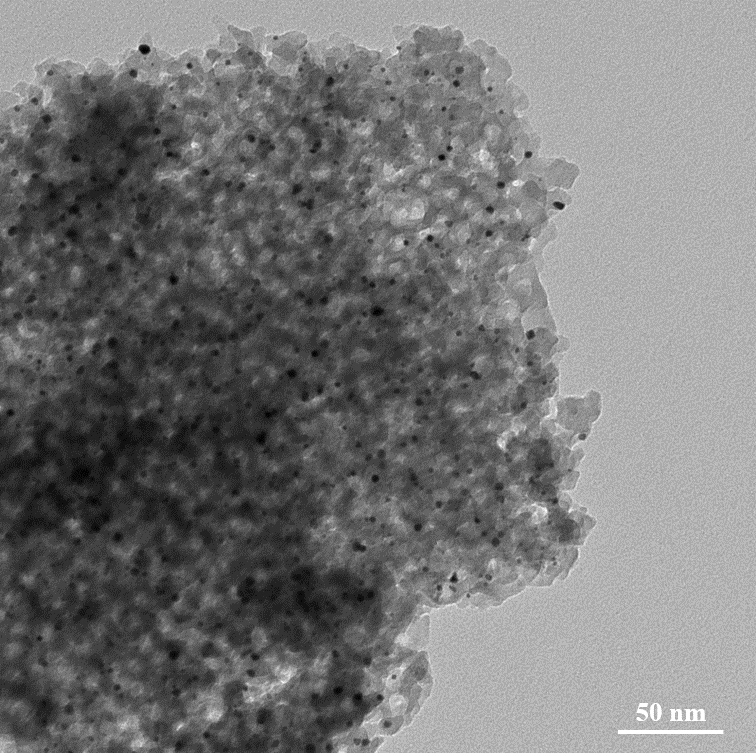


**Supplementary Figure 29.** TEM image of Au nanoparticles.


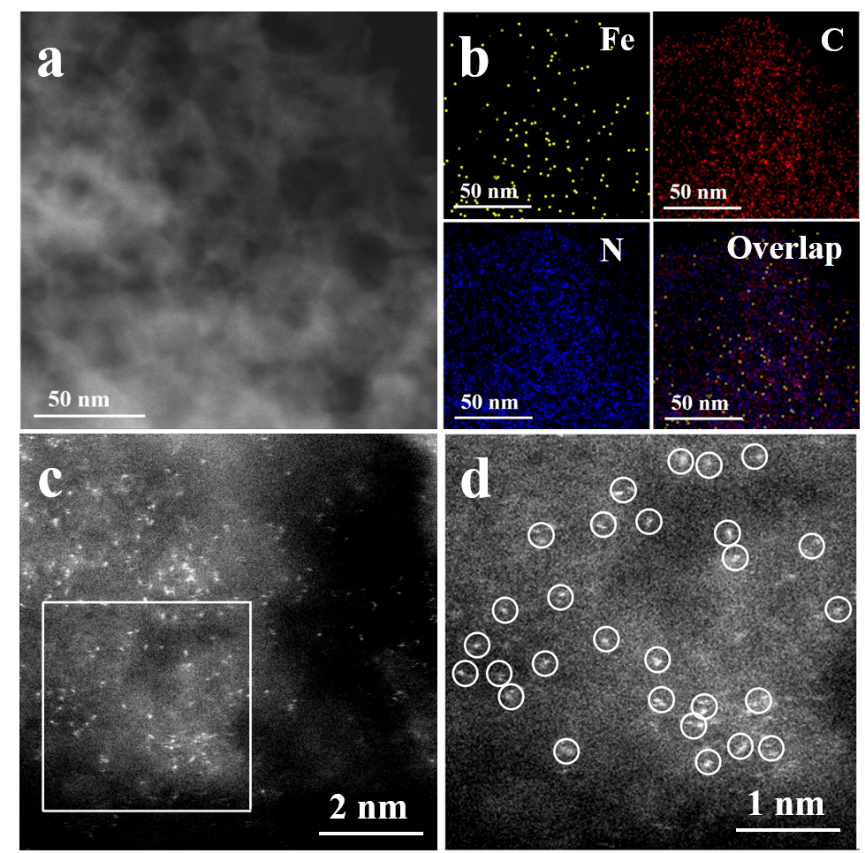


**Supplementary Figure 30.** a) HAADF-STEM images of Fe_2_/mpg-C_3_N_4_ after 15 times recycle. b) corresponding element maps showing distributions of Fe (green), N(red), C (blue), respectively. c) AC HAADF-STEM images of Fe_2_/mpg-C_3_N_4_. d) Magnified AC HAADF-STEM images of Fe_2_/mpg-C_3_N_4_ after 15 times recycle.


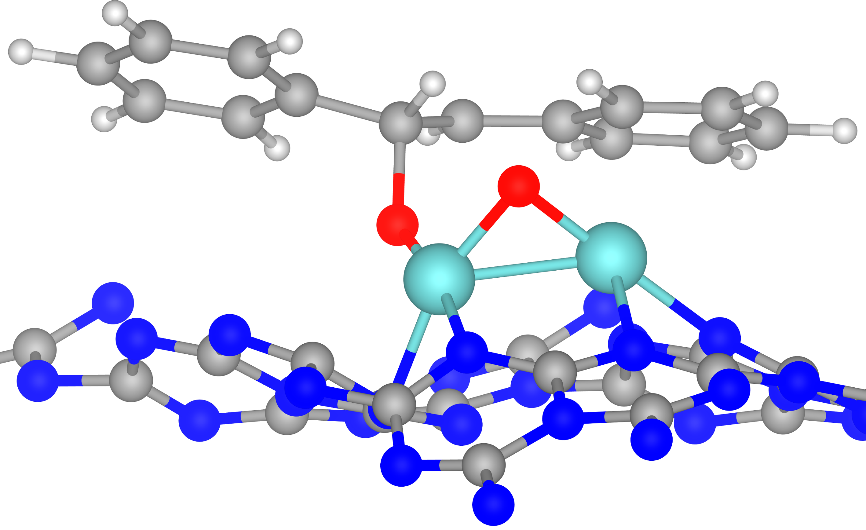


**Supplementary Figure 31**. Side view of the intermediate state in the *trans*-stilbene reaction pathway at the Fe_2_O_2_ site. The carbon, nitrogen, oxygen, hydrogen, and iron atoms are depicted in gray, blue, red, white, and cyan, respectively.


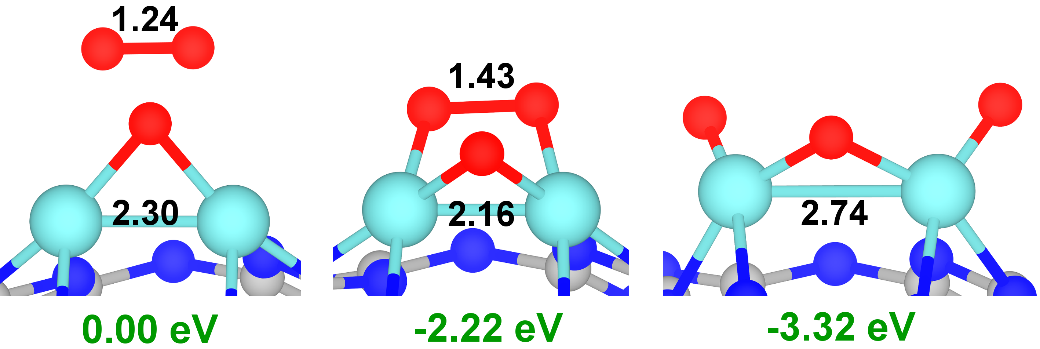


**Supplementary Figure 32**. Dissociative adsorption of an O_2_ molecule over the Fe_2_O site. Numbers in black represent the bond lengths (unit: Å), whereas whose in green refer to the energy changes. The formation of the molecular precursor (the middle panel) is barrierless, and the energy barrier of the subsequent O_2_ dissociation is less than 0.05 eV.


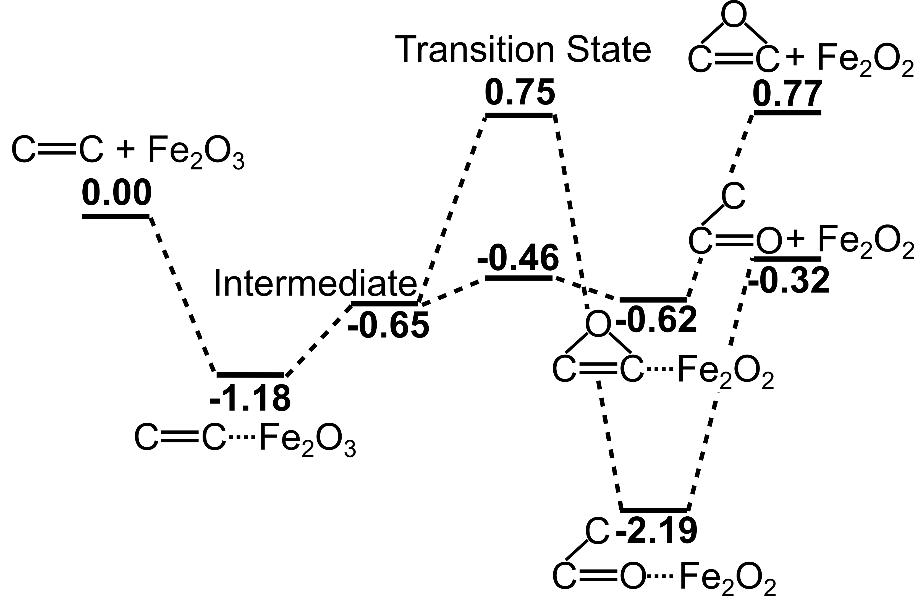


**Supplementary Figure 33**. Energy profile (unit: eV) for the *trans*-stilbene epoxidation at the Fe_2_O_3_ site.


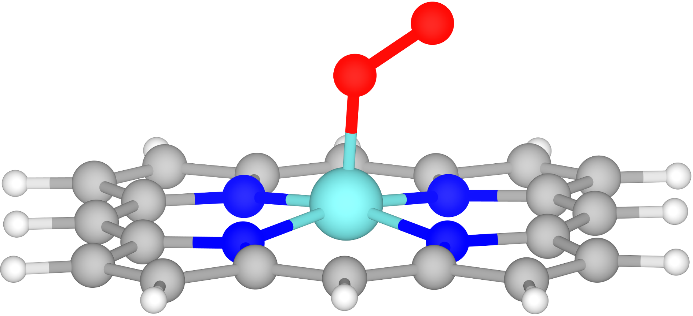


**Supplementary Figure 34**. Adsorption structure of an O_2_ molecule on the iron porphyrin substrate.


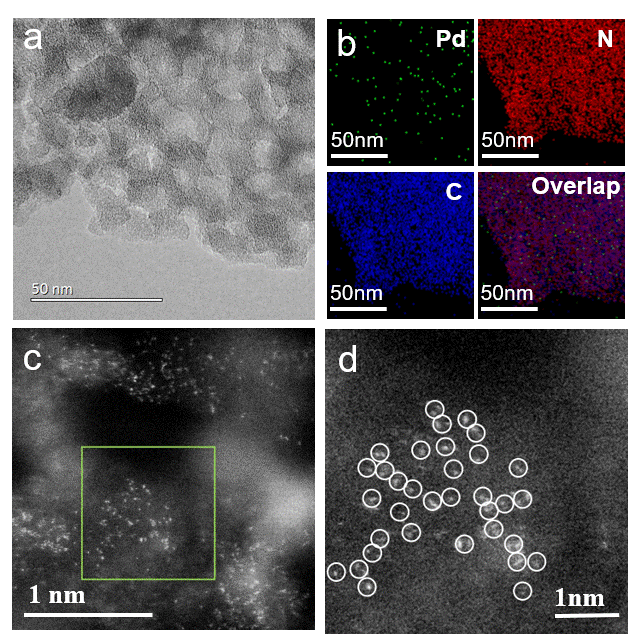


**Supplementary Figure 35.** a) HAADF-STEM images of Pd_2_/mpg-C_3_N_4_. b) corresponding element maps showing distributions of Pd (green), N(red), C (blue), respectively. c) AC HAADF-STEM images of Pd_2_/mpg-C_3_N_4_. d) Magnified AC HAADF-STEM images of Pd_2_/mpg-C_3_N_4_.


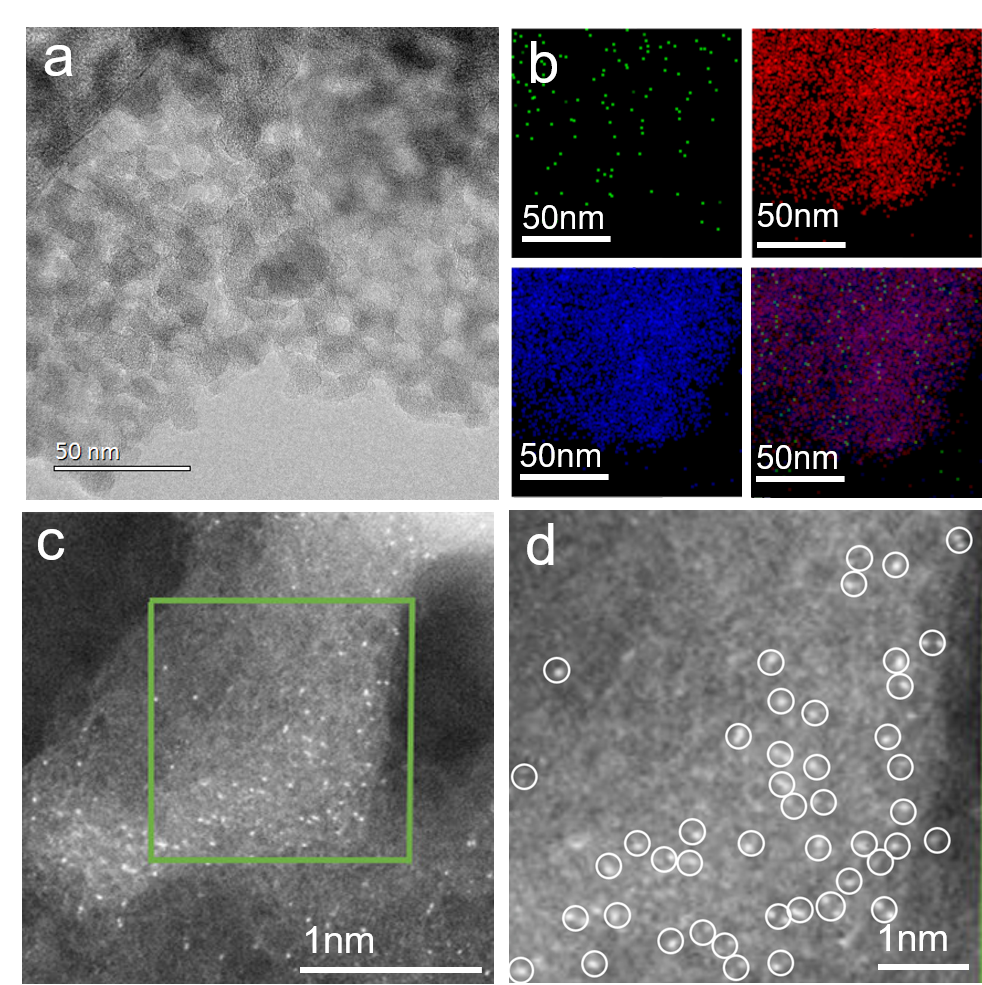


**Supplementary Figure 36.** a) HAADF-STEM images of Ir_2_/mpg-C_3_N_4_. b) corresponding element maps showing distributions of Ir (green), N(red), C (blue), respectively. c) AC HAADF-STEM images of Ir_2_/mpg-C_3_N_4_. d) Magnified AC HAADF-STEM images of Ir_2_/mpg-C_3_N_4_.

**Supplementary Table 3**. Structural parameters of Ir_2_-C_3_N_4_ and Pd_2_-C_3_N_4_ extracted from the EXAFS fitting. (S_0_^2^=0.85)

| Sample | Scattering pair | CN | R(Å) | σ^2^(10^-3^Å^2^) | ΔE_0_(eV) | R factor |
| --- | --- | --- | --- | --- | --- | --- |
| Ir_2_-C_3_N_4_ | Ir-N(O) | 3.6 | 1.98 | 3.1 | 2.5 | 0.0054 |
|  | Ir-Ir | 1.3 | 2.68 | 4.0 | 3.0 |  |
| Ir powder | Ir-Ir | 12* | 2.71 | 3.9 | 1.1 | 0.0062 |
| Pd_2_-C_3_N_4_ | Pd-N(O) | 3.4 | 1.97 | 4.5 | 1.5 | 0.0043 |
|  | Pd-Pd | 1.1 | 2.72 | 5.2 | 2.7 |  |
| Pd foil | Pd-Pd | 12* | 2.74 | 5.1 | 3.5 | 0.0032 |

S_0_^2^ is the amplitude reduction factor; CN is the coordination number; R is interatomic distance (the bond length between central atoms and surrounding coordination atoms); σ^2^ is Debye-Waller factor (a measure of thermal and static disorder in absorber-scatterer distances); ΔE_0_ is edge-energy shift (the difference between the zero kinetic energy value of the sample and that of the theoretical model). R factor is used to value the goodness of the fitting.

* This value was fixed during EXAFS fitting, based on the known structure of Ir powder and Pd foil.

Error bounds that characterize the structural parameters obtained by EXAFS spectroscopy were estimated as N ± 20%; R ± 1%; σ^2^ ± 20%; ΔE_0_ ± 20%.


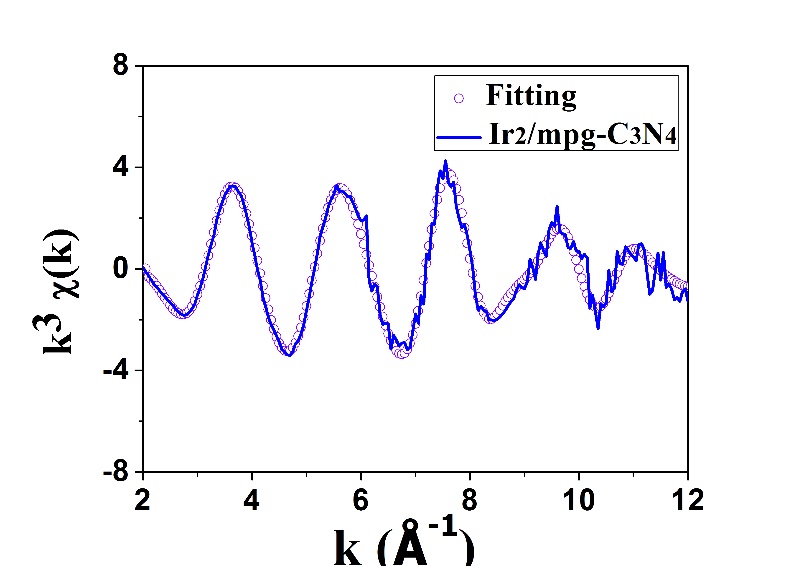


**Supplementary Figure 37.** EXAFS spectrum fitting curves of Ir_2_/mpg-C_3_N_4_ at k space.


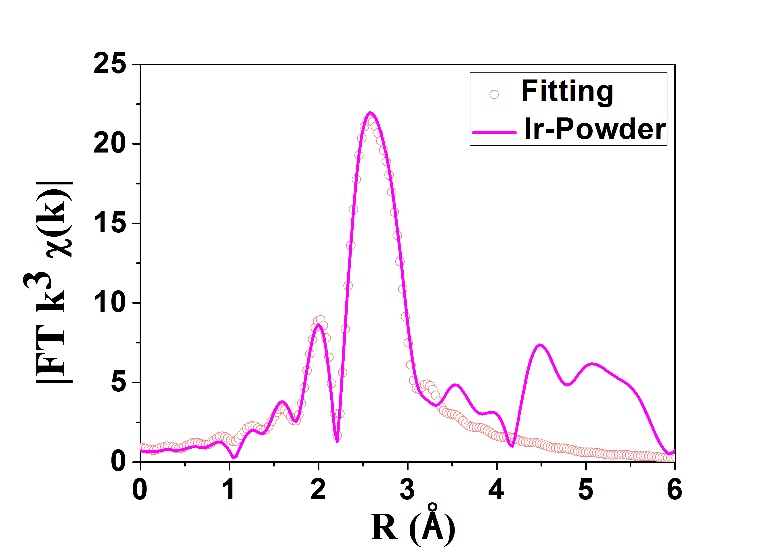


**Supplementary Figure 38.** EXAFS spectrum fitting curves of Ir_2_ Powder at R space.


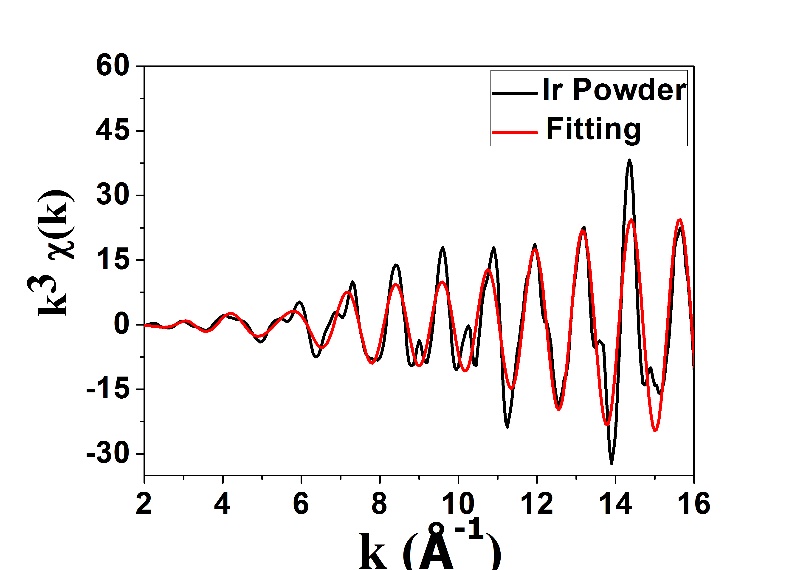


**Supplementary Figure 39.** EXAFS spectrum fitting curves of Ir_2_ Powder at k space.


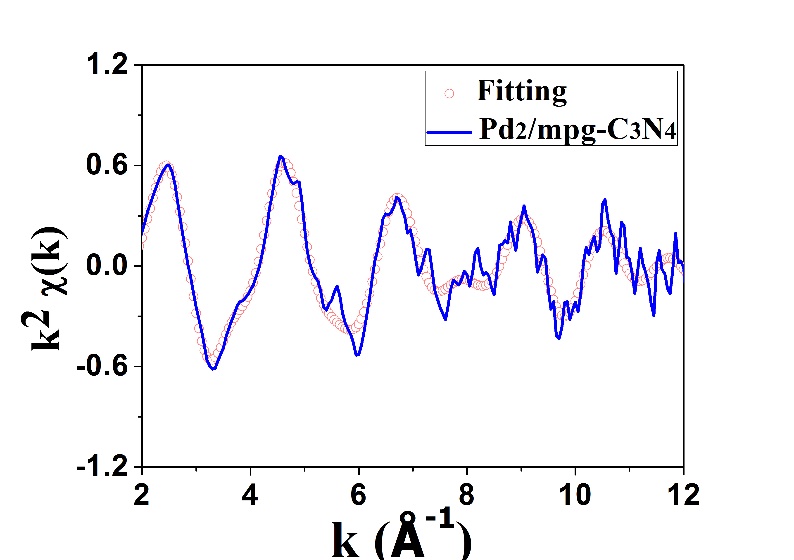


**Supplementary Figure 40.** EXAFS spectrum fitting curves of Pd_2_/mpg-C_3_N_4_ at k space.


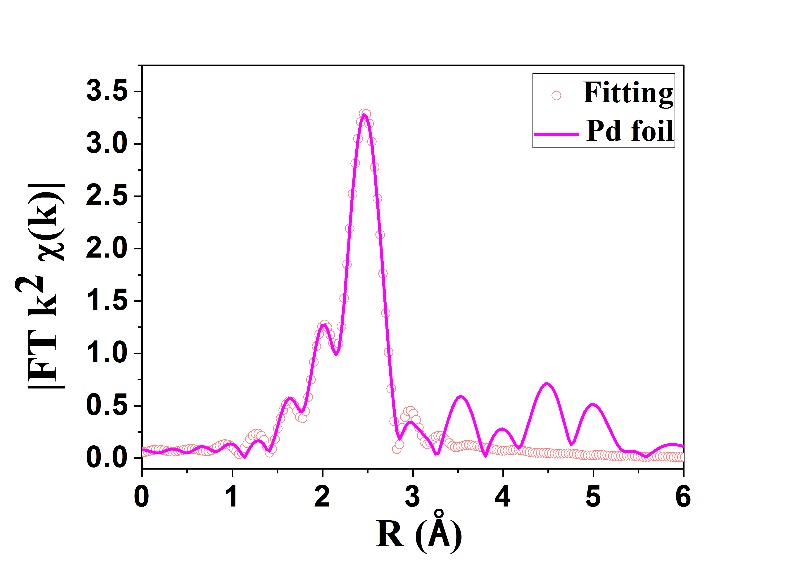


**Supplementary Figure 41.** EXAFS spectrum fitting curves of Pd foil at R space.


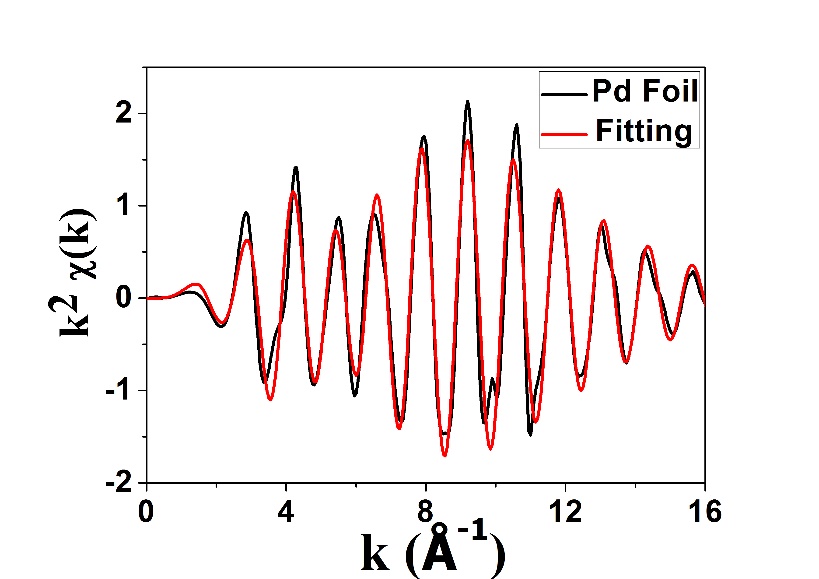


**Supplementary Figure 42.** EXAFS spectrum fitting curves of Pd foil at k space.





**Supplementary Figure 43.** TGA of bis(dicarbonylcyclopentadienyliron).

**Supplementary References**

1. Rehr, J. J. & Albers, R. C. Theoretical approaches to X-ray absorption fine structure. *Rev. Mod. Phys.* **72**, 621–654 (2000).

2. Joly, Y. X-ray absorption near-edge structure calculations beyond the muffin-tin approximation. *Phys. Rev. B* **63**, 125120 (2001).

3. Bunău, O. & Joly, Y. Self-consistent aspects of X-ray absorption calculations. *J. Phys. Condens. Matter.* **21**, 345501 (2009).
